# Awareness as a path to inner strength: a qualitative study on women living with heart failure

**DOI:** 10.3389/fcvm.2026.1744193

**Published:** 2026-10-20

**Authors:** Federica Dellafiore, Serena Cazzato, Flavia Primosa, Lucia Gotri, Orejeta Diamanti, Gloria Modena, Giovanna Artioli

**Affiliations:** 1 Department of Life Health Sciences and Health Professions, Link Campus University, Roma, Italy; 2 Local Health Service of Parma, Parma, Italy; 3 Department of Medicine and Surgery, University of Parma, Parma, Italy; 4 Healthcare Professions, Veneto Institute of Oncology IOV - IRCCS, Padua, Italy; 5 Welfare Services Area, IRCCS Istituto Clinico Humanitas, Rozzano, Milan, Italy

**Keywords:** experience, heart failure, inner strengths, qualitative research, women

## Abstract

**Background:**

Heart failure profoundly affects women's physical, emotional, and social well-being, often generating fear, uncertainty, and vulnerability that challenge adaptation and self-care. This study explored how women living with heart failure construct meaning and develop inner strength through illness awareness — defined as understanding the nature, severity, progression, and self-management implications of heart failure. Many women initially showed limited recognition of the seriousness and long-term trajectory of the condition, sometimes perceiving it as less threatening than other diagnoses (e.g., oncological conditions) and occasionally discontinuing treatment during periods of symptomatic improvement. The study also examined how awareness interacts with relationships and everyday adaptation.

**Methods:**

A qualitative design was applied using reflexive thematic analysis based on in-depth, semi-structured interviews with eleven women recruited from a cardiology research hospital in Northern Italy. Data were analyzed through a six-phase analytical framework to identify patterns of meaning and transformation.

**Results:**

Four interconnected themes described a developmental process of learning and adaptation: confronting vulnerability, reconnecting through others and faith, adapting daily life, and developing illness awareness and self-care. Participants revealed a gradual shift from emotional distress to reflective resilience, with awareness and self-reflection acting as catalysts for personal growth and empowerment. Limited illness awareness — particularly regarding disease severity, chronic progression, and the need for continuous treatment — conversely hindered adaptation and promoted passive coping.

**Conclusions:**

Developing illness awareness emerged as both an educational and therapeutic that fosters resilience, self-reflection, and active engagement in self-care. Integrating structured educational and psychosocial interventions into heart failure management may strengthen women's inner resources and enhance their capacity to live meaningfully with chronic illness.

## Background

Heart failure (HF) remains one of the most common and disabling chronic conditions worldwide, representing a major public health challenge and a leading cause of morbidity and mortality (1). Despite advances in pharmacological and device-based therapies, HF continues to be associated with a high symptom burden, frequent hospitalizations, and significant healthcare costs (2). In Europe, the incidence of HF is estimated at 3.2 cases per 1,000 person-years, with a prevalence of 17.2 per 1,000 individuals (1, 3). Among those affected, women account for nearly half of all cases and often live longer with symptomatic disease, multiple comorbidities, and poorer quality of life (4, 5). These clinical and psychosocial characteristics underscore the need for deeper exploration of how women experience and adapt to living with HF.

Women living with HF frequently describe profound emotional and existential challenges that accompany physical limitations. Feelings of fear, uncertainty, anxiety, social isolation, and loss of identity are common, reflecting how the illness reshapes daily life and relationships (6–8). Several studies have also highlighted limited disease awareness, poor illness perception, and fragmented understanding of self-care and treatment, which contribute to psychological distress and reduced adherence (9–11). In this context, HF can be interpreted not merely as a physiological condition but as a transformative life experience—a long learning trajectory in which individuals are called to reconstruct meaning, identity, and agency.

Developing disease awareness and health literacy plays a pivotal role in this adaptation. Awareness can be seen both as a prerequisite and as an outcome of a broader formative process that enables individuals to recognize symptoms, understand their condition, and take informed action (12). Through this process, patients may move from fear and uncertainty toward understanding, acceptance, and engagement. When awareness remains limited, however, passivity and emotional distress tend to prevail, revealing the importance of educational and supportive interventions that help patients make sense of their experience and strengthen self-management.

Within this experiential and educational trajectory, the concept of Inner Strength provides a meaningful theoretical framework to interpret how people grow through adversity. Originally developed from qualitative studies of women with chronic or life-threatening conditions, Inner Strength is defined as an internal developmental capacity that facilitates resilience, meaning-making, and adaptation (13, 14). It encompasses interconnected dimensions such as distress and search for meaning, connection with others and one's values, commitment and movement, and the construction of a new normal. Inner Strength enables individuals to transform vulnerability into growth and restore coherence in their lives. While the framework has been widely applied in oncology and other chronic illnesses, its relevance in women living with HF remains underexplored.

Despite the growing emphasis on patient-centred care, translating experiential knowledge into clinically meaningful strategies remains a persistent challenge in HF management. This is particularly relevant for populations whose illness experiences have been less extensively explored in depth, including women living with heart failure. Understanding how individuals interpret their illness, develop awareness, and mobilise personal resources is essential for designing educational and supportive interventions that are not only informative but also responsive to patients’ lived realities. Greater insight into women's experiential trajectories may help clinicians recognise how emotional responses, relational dynamics, and meaning-making processes influence symptom interpretation, help-seeking behaviours, and engagement in self-care. Such knowledge has practical implications for clinical communication, therapeutic education, and psychosocial support, enabling healthcare professionals to tailor interventions more effectively and support sustained self-management. By illuminating how awareness and Inner Strength develop within everyday life, this study contributes to bridging the gap between experiential understanding and the design of responsive, person-centred care strategies in heart failure. However, important gaps remain in understanding how these processes specifically unfold in women living with heart failure.

Despite these advances, important knowledge gaps remain. In particular, little is known about how Inner Strength develops in women living with heart failure and how it interacts with illness awareness throughout the adaptation process. A deeper understanding of these interrelated experiences may help identify experiential resources and barriers that can inform the development of responsive, person-centred educational and supportive interventions. Accordingly, this study aimed to explore how women living with heart failure develop Inner Strength during their illness trajectory and how disease awareness contributes to this process.

## Materials and methods

### Study design

This qualitative study aimed to gain a deep understanding of how women living with HF perceive and cope with their illness, with a specific focus on the development of inner strength and awareness as adaptive processes. Data were collected through semi-structured interviews and analyzed using Reflexive Thematic Analysis (RTA), following Braun and Clarke's approach (15, 16). RTA was chosen for its suitability in exploring subjective meanings, allowing for a flexible, interpretive engagement with participants’ narratives. The analysis and reporting process followed the Reflexive Thematic Analysis Reporting Guidelines (RTARG) (17), which provide structured recommendations for ensuring methodological transparency and coherence. The overall epistemological stance of this study was constructivist, recognizing that knowledge is co-constructed through interaction between researchers and participants (18).

### Sampling and setting

Participants were recruited using a purposive sampling strategy designed to include women able to articulate rich, reflective accounts of living with HF (19). The expression “able to articulate” refers to the capacity to communicate personal experiences verbally, engage in reflective dialogue during an in-depth narrative interview, and demonstrate adequate proficiency in the Italian language. Eligible participants were adult women aged 18 years or older, with a confirmed diagnosis of chronic HF for at least three months (2), fluent in Italian, and willing to participate in an in-depth interview. Exclusion criteria included severe cognitive impairment, acute clinical instability at the time of recruitment, or any condition that could significantly limit the ability to participate in an interview or provide informed consent. The focus on a female sample was theoretically justified to ensure conceptual coherence with the *Inner Strength* framework, which was originally developed to explore women's adaptation to chronic and life-threatening illnesses (13, 14). Investigating women with HF therefore offered a consistent context for examining how inner strength and awareness emerge in everyday life.

The study was conducted in the Cardiology Unit of a research hospital in Northern Italy. Clinic nurses informed all potentially eligible women attending routine outpatient visits about the study. They did not independently select participants but provided information to those who met the clinical inclusion criteria. Women who expressed interest were then contacted by a member of the research team, who verified eligibility, provided detailed study information, and obtained written informed consent. No financial or material incentives were offered for participation. This sample size was consistent with qualitative methodological guidance, which suggests that between eight and fifteen participants are sufficient to achieve information power and thematic sufficiency (20, 21).

Recruitment continued until the research team considered the dataset sufficiently rich, nuanced, and conceptually coherent to address the study aim and support meaningful interpretive thematic development. Consistent with reflexive thematic analysis, sample adequacy was not evaluated through coding redundancy or statistical representativeness, but through ongoing reflexive engagement with the depth, diversity, and interpretive potential of participants’ experiential narratives. The decision to conclude recruitment was therefore guided by thematic sufficiency and information richness rather than by positivist notions of data saturation (21).

### Data collection

Data were collected between November 2023 and January 2024 through semi-structured, in-depth interviews, a method suitable for exploring personal meanings, beliefs, and emotions (22) his approach was chosen to create a reflective space in which participants could articulate their lived experiences and gradually construct personal meaning around their illness. Interviews were conducted face-to-face at the hospital by a female nurse researcher with clinical and academic experience in cardiovascular nursing and prior training in qualitative interviewing and reflexive qualitative methodologies. The interviewer had previous experience conducting semi-structured interviews in healthcare research and was external to the participants’ direct clinical care team, with no prior therapeutic relationship with the women involved in the study. During selected interviews, other members of the research team, with backgrounds in nursing, healthcare education, and qualitative research, acted as observers and recorded contextual field notes and reflexive observations. Their role was limited to observational support and reflexive discussion and did not interfere with the interview process.

Field notes documented contextual features of the interview setting, salient non-verbal cues, and brief reflexive observations. Notes were drafted immediately after each interview and refined during transcription. They supported the analytic process by providing contextual depth, enhancing reflexivity, and contributing to data triangulation. The nurse researcher who conducted the interviews was external to the clinical care team and had no prior therapeutic relationship with the participants. Participation was entirely voluntary, and women were informed that declining or withdrawing from the study would not affect their care in any way.

Each interview began with an open, exploratory question—such as “*Can you tell me about the first time you heard about heart failure?”*—followed by prompts inviting participants to reflect on their emotional responses, coping strategies, relationships, and understanding of their illness. The interviewer adopted a dialogic stance, facilitating a narrative flow rather than a rigid question–answer format, allowing participants to move between past and present experiences. The interview guide covered five broad thematic areas related to the research aim (23): illness understanding and initial reactions, emotional impact, relationships and social support, lifestyle adaptations, and awareness of treatments and self-management.

All interviews were audio-recorded with participants’ consent and transcribed verbatim. To preserve participants’ anonymity, each interview was assigned a unique numerical identifier (e.g., Code 1, Code 2). These identifiers refer to anonymized interview transcripts and are used exclusively to attribute verbatim quotations throughout the Results section. They do not represent analytical codes generated during Reflexive Thematic Analysis. The average duration was 37 min, ranging from 30 to 45 min. Throughout the process, the interviewer used active listening, empathic reflection, and clarifying questions to support participants in exploring their inner perceptions and emotions. This reflexive interaction not only generated rich data but also fostered participants’ awareness of their own coping and adaptation processes. Interviews continued until each participant felt they had nothing further to add. Socio-demographic data were collected to describe the cohort. The interpretive and relational nature of the interviews aligned with the reflexive epistemology underpinning the thematic analysis, ensuring coherence between data generation and interpretive aims (Table 1).

**Table 1:** Interview guide and thematic areas.

Area of inquiry	Example questions and prompts
Opening question	Can you tell me about when and how you first became aware of your heart failure, and what that experience was like for you?
Emotions and meaning	• How did you feel when you first learned about your heart condition?
	• How have your emotions changed over time?
	• What helps you face moments of fear, uncertainty, or discouragement?
	• What does this experience mean to you now?
Self-perception and adaptation	• In what ways has living with heart failure changed the way you see yourself?
	• Have you discovered new strengths, abilities, or limits in yourself?
	• Can you describe an episode that helped you realize how you were adapting to your condition?
Relationships and connection	• How has heart failure influenced your relationships with family, friends, or healthcare professionals?
	• Who or what do you feel most connected to during difficult times?
	• How do these connections help you cope or find motivation?
Daily life and awareness	• How has heart failure affected your daily life and your way of managing it?
	• How do you recognize when your condition is changing or when you need help?
	• How do you take care of yourself on a daily basis?
Coping and personal growth	• What strategies or inner resources have helped you live with heart failure?
	• What gives you strength when you feel vulnerable or tired?
	• What have you learned about yourself through this experience?
Additional prompts	• How do you make sense of…?
	• What has this experience taught you?
	• How do you usually respond when you face a difficult day?
	• What keeps you going?
	• Can you tell me more about what helps you feel in control?
Closing question	Is there anything else about living with heart failure or about yourself that you would like to share or reflect on today?

### Data analysis

Interview transcripts were anonymized and analyzed using Reflexive Thematic Analysis following Braun and Clarke's six iterative phases (15, 16, 24). The process began with repeated readings to achieve familiarization with the data and the recording of reflexive memos. Two researchers independently performed inductive, line-by-line coding to identify units of meaning. These codes were then compared and discussed within the team to generate initial thematic patterns, which were subsequently refined into coherent, interpretive themes.

The coding process was iterative and reflexive. Transcripts were initially examined through inductive line-by-line coding to identify experiential, emotional, and relational meaning units relevant to the study aim. Early semantic and interpretive codes were progressively grouped into candidate thematic patterns through repeated comparison across interviews. These candidate themes were then refined, reorganised, and conceptually developed through ongoing reflexive dialogue within the research team, with attention to coherence, depth, and interpretive richness. The analysis moved recursively between data extracts, coding, thematic construction, and theoretical interpretation, consistent with Braun and Clarke's reflexive thematic analysis approach.

The analytic process was iterative and deeply interpretive, moving beyond description toward understanding how participants’ narratives revealed the gradual construction of awareness and inner strength. The team engaged in continuous reflexive dialogue to explore how their own professional perspectives influenced interpretation, viewing subjectivity as a resource that enriched meaning-making. Themes were continuously reviewed and adjusted to ensure they reflected participants’ lived experiences of heart failure, awareness, and the emergence of inner strength. Each theme was defined and named through collaborative discussion, and representative quotations were selected to illustrate participants’ voices. Consistent with RTA principles, the analysis emphasized meaning and depth rather than frequency; therefore, interrater reliability or coding counts were not reported, as these are not meaningful in reflexive qualitative research (16).

### Rigour and trustworthiness

Trustworthiness and methodological coherence were supported through strategies promoting credibility, transferability, dependability, and confirmability (25). Credibility was enhanced through iterative reflexive discussions within the research team, verbatim transcription, researcher triangulation, and a member reflection process conducted with a subset of participants. In line with reflexive thematic analysis, transcripts were not returned for factual verification; instead, four participants were invited to reflect on the resonance, clarity, and interpretive relevance of the preliminary thematic synthesis. Their feedback broadly confirmed the recognisability and meaningfulness of the themes, while also contributing to the reflexive refinement and interpretive nuance of selected thematic descriptions. Dependability was reinforced through the maintenance of an audit trail documenting analytic decisions, reflexive observations, and team discussions throughout the analytic process. To support confirmability, the researchers engaged in bracketing and reflexive journaling, critically examining preconceptions, professional assumptions, and emotional responses that could influence interpretation (26). Transferability was facilitated through detailed description of the research context, participants’ characteristics, and the interpretive boundaries of the findings.

Given the researchers’ professional backgrounds in nursing, cardiovascular care, and patient education, reflexive attention was continuously directed toward how prior clinical assumptions, educational perspectives, and emotional responses could shape interpretation. Rather than attempting to eliminate subjectivity, the research team approached reflexivity as an interpretive practice aimed at critically examining how meaning was co-constructed throughout the analytic process. Emerging interpretations were therefore regularly discussed, questioned, and refined through iterative reflexive dialogue within the multidisciplinary research team to support interpretive depth and thematic coherence.

### Ethical considerations

The study was conducted in accordance with internationally recognised ethical principles for research involving human participants, including those outlined by Emanuel et al. (27), and in compliance with the General Data Protection Regulation (EU 2016/679). The study consisted exclusively of voluntary, non-interventional qualitative interviews with competent adult participants and involved no experimental procedures, modification of clinical care, administration of treatments, or additional diagnostic procedures. According to the institutional procedures applicable at the study site and within the framework of the Regional Ethics Committee of Umbria (Comitato Etico Regionale dell’Umbria), this type of non-interventional qualitative inquiry did not require formal ethics committee review.

Before participation, all women received verbal and written information regarding the study aims, procedures, voluntary nature of participation, confidentiality safeguards, audio-recording procedures, anonymisation of data, and their right to withdraw at any time without consequences for their clinical care. Written informed consent was obtained from all participants prior to data collection and audio recording. To protect confidentiality and privacy, all interview transcripts were de-identified and stored in encrypted, password-protected files accessible only to the research team.

## Results

Eleven women participated in the study. The mean age of the participants was 79 ± 7.4 years. All had been living with heart failure for 8 ± 4.7 years, were retired, and presented with chronic heart failure. At the time of the study, participants’ functional status varied: six women were classified as NYHA class II, and five as NYHA class III, reflecting mild-to-moderate limitations in physical activity. The etiology of HF included ischemic cardiomyopathy (*n* = 5), hypertensive heart disease (*n* = 3), and valvular heart disease (*n* = 3). The analysis revealed four interrelated themes that together represent a transformative process through which women confronted, interpreted, and adapted to their illness. These themes reflect progressive dimensions of inner strength and awareness: confronting vulnerability, reconnecting through others and faith, adapting daily life, and learning through awareness and self-care (Table 2).

**Table 2:** Themes emerging from the reflexive thematic analysis.

Theme	Core idea	Representative quotation
1. Confronting vulnerability — fear, uncertainty, and the search for meaning	The initial stage of diagnosis is marked by existential fear and disorientation. Over time, women begin to search for meaning and re-establish a fragile sense of control.	“It felt like I couldn't handle it… The chaplain came, and I said: ‘Give me the last rites.’ Now I experience it differently because I’ve recovered; I feel good, I can walk without getting tired.” (codes 1, 11)
2. Reconnecting through others and faith	Emotional and spiritual connections with family, friends, and faith communities become essential resources, transforming isolation into belonging and dependence into reciprocity.	“My children even left their jobs to help me… I take things with patience, and the Lord watches over me from above.” (codes 5, 8)
3. Adapting daily life — between loss and redefinition	Daily routines and roles are reshaped by illness. Women mourn lost independence but slowly redefine normality, transforming restriction into resilience.	“I used to take long trips, visit exhibitions… but now I can't anymore. Now I can't do anything, I feel almost useless — yet they help a lot, even with cooking.” (codes 1, 4, 8, 10)
4. Learning through awareness and self-care	Awareness of illness and treatment emerges gradually through experience. Moving from unawareness to conscious engagement marks the final stage of adaptation and inner strength.	“I didn't fully realize the severity of the situation… Maybe it's my subconscious. Now I follow everything they prescribe and recommend.” (codes 3, 7)

### Theme 1: confronting vulnerability — fear, uncertainty, and the search for meaning

At the moment of diagnosis, women with HF vividly recalled a sudden rupture in their sense of safety and continuity, often occurring in the context of an acute symptom exacerbation leading to hospitalisation. The severity of physical symptoms—shortness of breath, profound fatigue, and swelling—contributed to a perception of imminent threat: “*I felt like I was dying*” (code 8). The language of “heart failure” itself reinforced feelings of fear and futility, as participants interpreted the term as indicative of a life-threatening, irreversible condition. These physical experiences were intimately linked to meaning-making: women described how bodily vulnerability prompted reflection on life priorities, relationships, and the seriousness of their condition. Over time, engagement with these physical cues helped transform fear into awareness and motivated steps toward adaptation and self-care. The awareness of being affected by a chronic, potentially life-threatening condition was described as a profound shock that shook both their bodies and their identities. “*It felt like I couldn't handle it,” one woman said, recalling the first encounter with her diagnosis* (code 11). Others relived the experience with the same intensity, as if the moment still lingered within them: “*The chaplain came, and I said: Give me the last rites*” (code 1). For several participants, this initial stage was marked by discouragement and sadness — “*I was a bit demoralized*” (codes 2, 11) — and by the sense that “*heart failure was a major blow*” (code 7).

As time passed, fear remained a pervasive presence but gradually evolved into a more reflective emotional state. Some participants described their ongoing fragility — “*I’m always afraid that it might happen again*” (code 12) — while others recognized the cyclical nature of anxiety: “*I still feel a bit uneasy and say to myself: What's happening to me?*” (code 9). Fatigue, apathy, and a lack of motivation were also frequent companions: “*Tired, apathetic, listless…”* (code 4). Yet within this vulnerability, small signs of acceptance began to appear. Some women found that by following medical recommendations and maintaining positive relationships with healthcare professionals, they could regain a sense of stability and control: “*Now I experience it differently because, in the end, I’ve recovered; I feel good, I can walk without getting tired, I can climb stairs, which I couldn't do before*” (code 11). Another participant echoed this regained balance: “*I feel well, that's for sure… they found me in pretty good condition because I follow everything they prescribe and recommend*” (code 3).

Across the interviews, women spoke of a tension between fear and resilience, loss and recovery. Their words reflected a process of meaning-making — a slow re-interpretation of illness that transformed fear into awareness and dependence into agency. While the experience of HF initially generated a deep sense of threat and existential anxiety, it also opened the possibility of re-evaluating life and health in new, more deliberate ways. This early stage of their journey corresponds to the first component of *inner strength* described by Dingley and Roux (13): the movement from distress toward the active search for meaning — a fragile but crucial step in the transformation of suffering into self-understanding.

### Theme 2: reconnecting through others and faith

After the initial shock of diagnosis, most women described an emerging awareness of the importance of connection — to others, to themselves, and to something larger than their illness. Although many experienced loneliness and a sense of isolation — “*I feel alone*” (codes 1, 5) — they also found renewed strength in the relationships that surrounded them. Family and friends became the scaffolding through which everyday life could be sustained, as one participant explained: “*My children even left their jobs to help me*” (code 8). Husbands, sometimes unexpectedly, took on caring roles that altered family dynamics: “*They help a lot… even with cooking*” (codes 2, 4). Others found comfort in the steadfast affection of sisters and daughters: “*I am well cared for by my family: my sisters and daughters. I love them, and they love me*” (code 5).

Support also extended beyond family. Neighbours and friends became essential companions in navigating the practical challenges of living with heart failure. “*I still live in a building where people are like they used to be. When I needed to go back and forth to the hospital, I always found someone to accompany me*” (code 9). These connections, built on reciprocity and affection, offered women not only tangible help but also a sense of belonging that counteracted their physical limitations and emotional vulnerability. Alongside human connection, many participants described a spiritual relationship that offered a parallel form of strength. Some spoke of cultivating an inner dialogue with themselves, drawing upon patience and serenity as moral resources: “*Serenity and patience, above all… patience*” (code 10). Others framed this endurance in explicitly religious terms: “*I take things with patience, and the Lord watches over me from above*” (code 5). Gratitude often accompanied this spiritual awareness. “*Now I say yes, thank God, I made it through*” (code 11), one woman reflected, while another acknowledged: “*He's the one who is still keeping me here”* […] and when “*He decides to take me, I will go*” (code 8).

While relationships provided strength, some women also described feelings of guilt, dependency, or burden when relying on others. These emotions sometimes generated tension or internal conflict, illustrating that connection could be both supportive and challenging. Recognizing this duality highlights the complexity of relational coping and underscores that even positive social interactions can require negotiation, acceptance, and adaptation. This connection — to family, friends, and the transcendent — nurtured the second dimension of inner strength: the discovery of self in relation to others. Through this reconnection, vulnerability began to coexist with gratitude, dependence with dignity, and fear with faith.

Faith, for these women, was not passive resignation but a way of giving meaning to suffering. It allowed them to situate illness within a broader moral horizon — to see it as part of life's journey rather than an interruption of it. As one participant summarized, “*I’m quite strong. I react; I don't turn it into a tragedy*” (code 7). Together, these relational and spiritual resources strengthened participants’ capacity to adapt, illustrating how connection became a central component in the development of inner strength.

### Theme 3: adapting daily life — between loss and redefinition

For many women, HF imposed a clear boundary between a before and an after. Life prior to the illness was recalled with nostalgia — a time when their bodies allowed freedom, autonomy, and pleasure. After diagnosis, everyday routines required radical reorganization, often accompanied by a painful sense of loss. As one woman said, “*I used to take long trips… visit exhibitions… but now I can't anymore, because staying in the car makes me feel unwell*” (code 1). Others mourned the loss of simple but meaningful activities: “*Cooking… or gardening… I can't handle the physical strain anymore*” (code 7). Changes in diet were another tangible reminder of constraint: “*I love eating cured meats, but we’ve had to eliminate them… my diet is definitely poorer compared to before*” (codes 1, 2). This forced adaptation generated frustration and grief. “*Now I can't do anything*,” one participant admitted (code 10). Another confessed, “*I feel almost useless*” (code 8). The loss of autonomy often carried emotional and social repercussions: “*I can't do what I want, I can't go out alone. Before, I used to go out and about, I didn't need my husband… If I had errands to run, I would just go*” (code 10). For many, this transition produced a sense of dependence and an inner struggle to accept new physical and social limitations.

Yet even amid these losses, subtle forms of redefinition emerged. Some women described a process of negotiating new rhythms and boundaries, recognizing that “doing less” did not necessarily mean “being less.” A few began to appreciate the care and attention they received from others, interpreting it not as dependence but as reciprocity: “*They help a lot… even with cooking*” (code 4). Others found solace in the routines that restructured their days — medication schedules, short walks, or quiet moments at home — transforming these necessities into symbols of endurance.

This theme illustrates the *movement* component of inner strength described by Dingley and Roux (13): the capacity to shift from resistance to adaptation, from lamenting loss to redefining normality. The women's stories reveal a slow, fragile reconstruction of self — a balancing act between the body's limitations and the mind's determination to re-establish meaning and control. Through this process, daily life becomes both the arena of constraint and the space where a new form of agency quietly takes root.

### Theme 4: learning through awareness and self-care

Although most women had lived with HF for several years, many continued to describe an uncertain and fragmented understanding of their illness. By awareness, we refer specifically to participants’ understanding of their disease severity, progression, and implications for self-management. Awareness emerged as a fragile, sometimes fluctuating dimension — a slow, uneven learning process rather than a stable acquisition of knowledge. Several participants openly acknowledged their difficulty in grasping the meaning and seriousness of their condition: “*I do not fully realize the severity of the situation*” (code 7); “*Maybe it's my subconscious*” (code 7). Others expressed a kind of emotional detachment, as if the disease still belonged to someone else's story: “*I had heard about it before, but distantly, as if it didn't concern me*” (code 4).

This limited awareness often translated into challenges in recognizing bodily signals or adhering to treatment. Some women admitted uncertainty about the rationale behind medications: “*I don't know how to take the medication, even though I know I have to take care of myself*” (code 7). Others illustrated how this partial understanding led to risky decisions: “*The doctor said otherwise I could have a stroke… So I just take them, that's it*” (code 10). One woman described stopping her therapy during periods of symptomatic improvement because she “wasn't eating much,” adding: “*I was doing fine… but then I suddenly couldn't breathe, and they had to take me to the ER*” (code 5). These experiences reveal a gap between knowledge and action, showing how limited recognition of the chronic trajectory of HF can compromise adherence and self-care.

Yet, within these limitations, subtle learning processes were visible. For some women, repeated hospitalizations, conversations with healthcare professionals, and reflection on their own experiences gradually increased their understanding, transforming passive compliance into conscious engagement. “*Now I experience it differently because I follow everything they prescribe and recommend*” (code 3). Others, though still uncertain, began to recognize the connection between their daily behaviors and well-being. Even the simple act of “*keeping going*” (codes 1, 5, 7, 10) — once a passive expression — began to take on a new connotation of persistence, agency, and hope.

This final theme reflects the new normal phase of inner strength, where illness awareness and active self-care merge into a more deliberate approach to living with heart failure. The women's narratives suggest that awareness is not a given, but a form of personal education developed through experience, reflection, and relational support. Their gradual movement from limited recognition and inconsistent adherence to conscious engagement represents the final stage of the inner strength process: a fragile, evolving equilibrium in which vulnerability and knowledge coexist, enabling women to transform mere survival into a conscious, meaningful way of living with their chronic condition.

## Discussion

This study explored the lived experiences of women with HF, focusing on the emotional, relational, and practical dimensions of their condition. The findings illuminate how women gradually develop processes of awareness and inner strength, moving from fear and uncertainty toward adaptation and meaning. The four themes—confronting vulnerability, reconnecting through others and faith, adapting daily life, and learning through awareness and self-care—represent a developmental sequence that mirrors the evolution of inner strength as described by Dingley and Roux's theoretical model (13). Importantly, this sequence can also be interpreted as an experiential learning pathway, through which women progressively acquire knowledge, emotional literacy, and coping competence in managing their chronic condition.

The first stage, confronting vulnerability, revealed the existential rupture women experienced at diagnosis. Participants described fear, loss of control, and a sense of mortality—emotions that disrupted their sense of continuity and identity. Statements such as “It felt like I couldn't handle it” and “I felt like I was dying” expressed both the intensity of this moment and the fragility that followed. According to Dingley and Roux (13), distress represents the gateway to inner strength, as it creates the emotional tension that can lead to transformation. From an educational perspective, this moment of crisis can be seen as a “threshold” for learning—an affective turning point that, if properly supported, can foster insight and motivation for self-management. However, our participants often remained suspended in this initial state of distress for a prolonged time, suggesting that the activation of inner strength may require additional support mechanisms—particularly structured educational and psychosocial interventions that help women make sense of their illness, develop self-awareness, and reframe vulnerability as a starting point for learning rather than defeat.

The second theme, reconnecting through others and faith, corresponds to the “connection” component of the Inner Strength Theory. The women's narratives underscored the importance of relational and spiritual resources as anchors of stability. Family members, friends, and neighbors were described as sources of tangible and emotional support, transforming loneliness into belonging and dependence into reciprocity: “My children even left their jobs to help me,” one woman explained. Similarly, spirituality emerged as a key coping mechanism—“I take things with patience, and the Lord watches over me”—offering comfort, acceptance, and renewed purpose. These findings align with prior evidence showing that inner strength in women with chronic illness often arises through relational and spiritual connectedness (28). From a cardiovascular education standpoint, connection appears not only as a protective factor but as a relational learning context in which women co-construct meaning and resilience. Health professionals—especially nurses, psychologists, and social workers—can play an active educational role in facilitating these supportive networks, promoting peer dialogue, and acknowledging spiritual needs as legitimate components of holistic care.

The third theme, adapting daily life—between loss and redefinition, represents the “movement” or “commitment” phase of inner strength (13). The women described a painful reorganization of their daily routines, marked by the loss of previously valued activities: “I used to take long trips, visit exhibitions… but now I can't anymore.” Feelings of frustration, uselessness, and dependence were frequent—“Now I can't do anything… I feel almost useless”—yet some participants began to reinterpret these limitations as opportunities for renewal, transforming routine care practices into signs of resilience and control. This adaptive process mirrors experiential learning, in which daily challenges become opportunities for reflection, experimentation, and the reconstruction of meaning. Consistent with findings by Yang et al. (29), adaptation in women with HF can be viewed as a dynamic learning process, where awareness grows through the reconfiguration of habits and relationships (30). Educationally, this suggests that nursing interventions should integrate reflective dialogue, goal-setting, and coaching techniques to help patients translate awareness into new life skills and sustainable behavioral change.

The final theme, learning through awareness and self-care, highlights that awareness in HF is not static but developmental. Many women described limited understanding of their illness—“I do not fully realize the severity of the situation”—or an automatic, unreflective adherence to treatment: “I just take them, that's it.” These statements reveal a gap between doing and understanding. However, some women described a shift toward conscious engagement: “Now I follow everything they prescribe and recommend.” This progression illustrates how self-care literacy and reflective awareness can foster autonomy and strengthen inner capacity. Research has demonstrated that low health literacy is common among patients with HF and is associated with poorer outcomes (31); conversely, structured education improves adherence, empowerment, and quality of life (32). Our findings expand this evidence by showing that awareness is not merely cognitive—it is existential and emotional, tied to meaning-making and identity reconstruction. Promoting awareness thus becomes both an educational and therapeutic act—one that integrates knowledge, reflection, and empathy, and transforms passive adherence into empowered engagement.

Our findings suggest that illness awareness develops alongside inner strength rather than as a separate process. Women described a gradual transition from fear and uncertainty to a better understanding of their condition, supported by lived experience, relationships, and interactions with healthcare professionals. As awareness increased, participants also described greater confidence in managing daily life and engaging in self-care. These findings suggest that patient education in heart failure should not focus exclusively on providing information but should also support reflection, dialogue, and the interpretation of personal illness experiences. Educational interventions that acknowledge emotional, relational, and experiential dimensions may help women integrate self-care into everyday life and strengthen their capacity to adapt to living with heart failure. This interpretation is consistent with recent literature emphasizing the importance of integrating educational and psychosocial approaches within cardiovascular nursing practice (33, 34).

### Gendered implications

Although many of the experiences described by participants—such as vulnerability, loss of control, disruption of identity, and existential uncertainty—have been widely documented among people living with HF regardless of gender, our findings suggest that these processes take on specific meanings when situated within women's life contexts. The present study does not indicate that such experiences are exclusive to women; rather, it highlights how they are interpreted, negotiated, and integrated in ways that reflect socially and relationally structured roles and expectations. For many participants, the illness challenged not only physical functioning but also long-standing relational identities, including caregiving responsibilities, family roles, and expectations of emotional availability. The disruption of these roles often intensified the sense of existential rupture, as limitations imposed by HF were experienced not only as personal losses but also as perceived failures to maintain relational continuity. In this sense, vulnerability was not solely embodied but relationally mediated.

Similarly, the development of illness awareness and self-care appeared closely intertwined with relational dynamics. Participants frequently described learning to recognize bodily signals, accept limitations, and engage in treatment through interactions with family members, healthcare professionals, and significant others. Inner strength therefore emerged not only as an individual psychological resource but as a relationally embedded process shaped by responsibility, connection, and meaning-making within everyday life. These findings suggest that while the existential impact of HF may be broadly shared across genders, the pathways through which individuals reconstruct identity, develop awareness, and engage in self-management are shaped by lived social positions and relational commitments. Attending to these contextual influences does not imply essential gender differences but rather supports a more nuanced understanding of how illness adaptation unfolds in real-life settings. From a clinical perspective, this highlights the importance of recognising relational and contextual dimensions of coping when supporting women living with heart failure. Educational and supportive interventions may benefit from addressing role renegotiation, relational expectations, and the gradual development of illness awareness as part of a broader adaptive process. Such an approach may enhance the capacity of healthcare professionals to provide person-centred care that is responsive not only to symptom burden but also to the lived structures that shape patients’ engagement with illness.

### Limitations and practical implications

As with all qualitative inquiries, the purpose of this study was not to generalize but to provide a rich, contextualized understanding of women's lived experiences with heart failure. The findings should thus be interpreted within their specific sociocultural and clinical context. Data were collected from a purposive sample of eleven women attending a single cardiology research hospital in Northern Italy. While this setting offered coherence and depth, it may also have influenced participants’ perspectives and coping styles. Transferability to other contexts should therefore be approached cautiously. Furthermore, educational level, socioeconomic status, and living arrangements were not systematically collected. Consequently, the influence of these contextual factors on participants’ experiences and perceptions of living with heart failure could not be explored. Future qualitative research should consider incorporating these variables to provide a more comprehensive understanding of the sociocultural context in which illness experiences and adaptation processes unfold. In addition, although NYHA functional class and heart failure etiology were available for all participants, ACC/AHA heart failure staging was not routinely recorded, limiting the possibility of further contextualizing participants’ disease progression.

The cross-sectional nature of the study represents another limitation. Since interviews were conducted at one point in time, it was not possible to observe the evolution of inner strength and awareness as dynamic and time-dependent processes. Future longitudinal or serial-interview studies could provide a more comprehensive understanding of how these constructs develop throughout the HF trajectory. Reflexivity is a further element of consideration. Although the research team applied bracketing, member checking, and group discussion to ensure rigor, the investigators’ nursing background may have influenced the interpretative lens. Future research would benefit from interdisciplinary collaborations among nurses, psychologists, and patient educators, or participatory approaches involving women with HF as active contributors to educational tool design and evaluation.

Despite these boundaries, the study yields significant practice implications. The findings emphasize the importance of integrating gender-sensitive, person-centered educational interventions that acknowledge emotional vulnerability as a legitimate component of the HF experience. Recognizing fear and uncertainty at the early stages of care could facilitate the activation of inner strength and prevent maladaptive coping. Similarly, relational and spiritual connections emerged as potent sources of resilience and should be considered integral to care planning. Supporting lifestyle adaptation must go beyond physical rehabilitation to include reflective guidance that helps women reconstruct identity and meaning in daily life. Finally, promoting awareness should be conceived not as the transfer of information but as a shared learning process—a compassionate educational act that transforms vulnerability into self-knowledge and resilience. By embedding these educational, relational, and multidisciplinary dimensions within cardiovascular nursing practice, healthcare professionals can help women with HF move from chronic vulnerability toward greater awareness, autonomy, and well-being.

## Conclusions

This study provides an in-depth understanding of how women with HF experience and make sense of their illness, revealing that inner strength and awareness evolve as intertwined, dynamic processes. The findings suggest that many women navigate fear, dependency, and loss before gradually achieving self-reflection and adaptation. Awareness emerges as both an educational process that enables this transformation, turning passive compliance into conscious, empowered self-care. From a clinical and educational perspective, promoting awareness should be viewed as a core nursing competence—a process of guided reflection that helps patients attribute meaning to their condition and reconstruct a sense of control. Integrating these approaches within cardiovascular care could enhance women's psychological resilience, strengthen adherence, and improve long-term outcomes. Future research should further examine how educational and relational interventions can activate inner strength, particularly through longitudinal and participatory designs that capture its evolution over time.

## Data Availability

The raw data supporting the conclusions of this article will be made available by the authors, without undue reservation.
